# Eotaxin-1 in exhaled breath condensate of stable and unstable asthma patients.

**DOI:** 10.1186/1465-9921-11-110

**Published:** 2010-08-12

**Authors:** Ziemowit Zietkowski, Maria M Tomasiak-Lozowska, Roman Skiepko, Elzbieta Zietkowska, Anna Bodzenta-Lukaszyk

**Affiliations:** 1 Department of Allergology and Internal Medicine, Medical University of Bialystok, Poland; 2 The Teaching Hospital of the Medical University of Bialystok, Poland

## Abstract

**Background:**

Airway eosinophilia is considered a central event in the pathogenesis of asthma. Eotaxin plays a key role in selective eosinophil accumulation in the airways and, subsequently, their activation and degranulation. The study was undertaken to evaluate eotaxin-1 levels in the exhaled breath condensate (EBC) of asthmatics with different degrees of asthma severity and to establish the possible correlation of these measurements with other recognized parameters of airway inflammation.

**Methods:**

EBC was collected from 46 patients with allergic asthma (14 with steroid-naïve asthma, 16 with ICS-treated, stable asthma, 16 with ICS-treated unstable asthma) and 12 healthy volunteers. Concentrations of eotaxin-1 were measured by ELISA.

**Results:**

In the three groups of asthmatics, eotaxin-1 concentrations in EBC were significantly higher compared with healthy volunteers (steroid-naïve asthma: 9.70 pg/ml ± 1.70, stable ICS-treated asthma: 10.45 ± 2.00, unstable ICS-treated asthma: 17.97 ± 3.60, healthy volunteers: 6.24 ± 0.70). Eotaxin-1 levels were significantly higher in patients with unstable asthma than in the two groups with stable disease. We observed statistically significant correlations between the concentrations of eotaxin-1 in EBC and exhaled nitric oxide (F_ENO_) or serum eosinophil cationic protein (ECP) in the three studied groups of asthmatics. We also discovered a significantly positive correlation between eotaxin-1 in EBC and blood eosinophil count in the groups of patients with unstable asthma and steroid-naïve asthma.

**Conclusions:**

Measurements of eotaxin-1 in the EBC of asthma patients may provide another useful diagnostic tool for detecting and monitoring airway inflammation and disease severity.

## Introduction

Asthma is a chronic inflammatory disease of the airways. Eosinophils play a crucial role in the pathogenesis of asthma, and eosinophil infiltrations prevail in sites of allergic inflammation. The most important factors taking part in the development of inflammatory infiltration are increased expression of adhesion molecules localized on the surface of endothelial cells (VCAM-1 - vascular cell adhesion molecule-1) and increased synthesis of chemotactic substances by eosinophils and Th2 lymphocytes [1,2]. Many factors are known which increase eosinophil chemotaxis to the site of inflammation as well as prolonging their survival, e.g. IL-3, IL-5, GM-CSF (granulocyte monocyte colony stimulating factor) [3]. They act together with selective chemokines of eosinophils, such as eotaxin, RANTES or MCP-4 (monocyte chemotactic protein-4). The strongest and the most specific chemoattractant is eotaxin [1,2].

In previous studies it was revealed that eotaxin levels in BAL fluid were higher in asthmatics than in healthy controls [4,5]. Eotaxin concentrations in sputum were also significantly raised in moderate and severe asthma patients when compared with healthy controls [6]. However, these relatively invasive approaches are unsuitable for repeated monitoring of airway inflammation.

By contrast, exhaled breath condensate (EBC), collected by cooling exhaled air, is a noninvasive, easily performed, and rapid method for obtaining samples from the lower respiratory tract. There has been increasing interest in measuring EBC, which is a very useful method in the pathophysiology and evaluation of new strategies for the treatment of asthma - especially for the assessment of inflammatory mediators related to the bronchial epithelium [7]. However, the analysis of EBC is still in its experimental phase and there remain many methodological questions in this method [8].

Previous studies have revealed that eotaxin can be measured in the EBC of patients with asthma [9-11]. Ko et al reveal that eotaxin levels are higher in asthmatics treated with inhaled steroids than in steroid-naïve asthmatics or healthy controls. The authors suggest that exhaled chemokines may be potential non-invasive markers for assessing airway inflammation in asthmatics [9]. However, to confirm such usefulness for eotaxin, the establishment of correlations of its levels with other recognized markers in the assessment of eosinophilic inflammation is needed. Such data are not available so far in published studies.

We hypothesize that eotaxin-1 levels in EBC are associated with the level of severity of the disease and markers of airway inflammation in asthma.

The aim of the study was to assess eotaxin-1 concentrations in the EBC of asthmatics with different degrees of asthma severity, and also to establish the possible correlation of these measurements with the other recognized parameters of airway inflammation.

## Material and methods

### Study population

The study was performed upon groups of 14 steroid-naïve allergic asthma patients; 16 patients, treated with inhaled corticosteroids (ICS) with stable allergic asthma; and 16 ICS-treated patients with unstable allergic asthma. Asthma was diagnosed according to the criteria recommended by the GINA 2006 [12].

The steroid-naïve asthmatics had not been treated with ICS. They were free from acute exacerbations and respiratory tract infections during the three months prior to the study. In this group of patients asthma was diagnosed recently, just before inclusion to the study (on the basis of symptoms and other recognized tests, such as reversibility test or/and bronchial provocation tests).

The patients with unstable ICS-treated asthma and stable ICS-treated asthma had a many-year history of asthma and anti-asthmatic treatment. In these patients asthma was diagnosed several years ago on the basis of typical symptoms, positive reversibility tests, or bronchial provocation tests. The patients with stable ICS-treated asthma had been treated with low to medium doses of ICS at a constant dose for at least three months. Stable asthma was defined as a minimal need for rescue medications (short-acting β_2_-agonists), no exacerbations, and no use of systemic steroids in the previous 12 months. The patients with unstable asthma had required one or more hospitalizations for asthma and more than three oral steroid bursts in the last year. They had been taking high-doses of ICS and long-acting β_2_-agonists for at least six months. Patients who had respiratory tract infections in the last month before the study were excluded from this study. All the patients were atopic and sensitized to common perennial inhaled allergens, as evaluated by skin prick tests (with commonly encountered aeroallergens: house dust mites, trees, weeds, grasses, cat, dog, Alternaria and Cladosporium).

12 healthy subjects were recruited for the study as a negative control group. In this group, asthma was excluded on the basis of lack of symptoms of asthma and atopy, normal spirometric indices, low exhaled nitric oxide (F_ENO_) levels, and no presence of eosinophilia in peripheral blood. Healthy volunteers also had a negative bronchial provocation test with histamine (PC_20 _> 32 mg/ml). All healthy volunteers were non atopic; all of them had negative skin prick tests. They were free of respiratory tract infection within three months prior to the study and from other significant illnesses known to affect F_ENO _measurements. Asthma patients and healthy volunteers were non-smokers and during the last year had not been passive smokers.

### The scheme of the procedures during the study

After inclusion to the study, the history of every patient with asthma was taken, then all patients were examined by the physician and blood (to determine serum total IgE, ECP, and blood eosinophil count) and EBC samples were collected. After 30 min, the measurement of F_ENO _level and spirometry were performed. Subsequently, all patients had skin prick tests. In healthy volunteers, all these procedures were carried out in the similar sequence. Finally, in all studied healthy subjects, a non-specific bronchial provocation test with histamine was performed.

The study protocol was approved by the Ethics of Research Committee of the Medical University of Bialystok, number of agreement: R-I-002/265/2009. Informed consent was obtained from every patient entered in the study.

### Exhaled nitric oxide measurements

Exhaled nitric oxide (F_ENO_) was measured by the chemiluminescence technique using a Sievers 280i NO Analyzer (Boulder, Colorado, USA). The measurements were performed at an expiratory flow of 50 ml/s according to ATS recommendations for on-line measurement of F_ENO _in adults [13].

### Lung function

The spirometry (FEV_1_) was performed using a MasterScreen Pneumo PC spirometer (Jaeger, Hoechberg, Germany), according to ATS standards [14].

### Collection of exhaled breath condensate

EBC was collected by using a commercially available condenser (EcoScreen; Erich Jaeger GmbH, Hoechberg, Germany) according to the current ATS/ERS guidelines [7]. All measurements were performed at the same time (between 8.00-10.00 am) to avoid possible circadian rhythm of mediator concentrations in EBC. All patients were asked to refrain from eating and drinking before collecting EBC. Exhaled air entered and left the chamber through one-way valves and an inlet and outlet, thus keeping the chamber closed. A low temperature inside the condensing chamber throughout the collection time produced a cooling down sample. The temperature of collection was around 0°C [7,15]. Patients were instructed to breathe tidally for 10 minutes with nose clip. The respiratory rate ranged from 15-20 breaths/minute. Patients were asked to swallow their saliva periodically and to temporarily discontinue collection if they needed to cough. At the end of collection 1.5 to 3.5 ml aliquots of condensate were transferred to Eppendorf tubes and immediately frozen. Samples were stored at -80°C [16].

The longest storage time of EBC samples did not exceed two months. The samples were not concentrated prior to measurement. All measurements were performed in a blind fashion. All samples were run in duplicate. Because the marker used to correct the difference in the degree of dilution has not yet been established, in our study we made no attempt to assess the dilution of ALF in EBC. The results (eotaxin-1) were well repeatable {CV(%) = 4-7%}. We performed the preliminary study, in which we measured eotaxin-1 in EBC immediately after collection and after 1, 2, and 3 months of storage at -80°C and we did not observe important changes. Therefore, we suggest that eotaxin-1 in EBC stored at -80°C remains stable during at least three months.

### Measurements of eotaxin-1, ECP and other laboratory parameters

Serum total IgE concentrations and serum ECP were measured using ImmunoCAP™ Technology (Pharmacia Diagnostics, Uppsala, Sweden). The minimum detectable level of ECP was 2.0 μg/l. Blood eosinophil count was measured using a hematologic analyzer (Coulter Electronics GmbH, Miami, Florida, USA). The concentrations of eotaxin-1 (R&D Systems, Wiesbaden-Nordenstadt, Germany) in EBC were determined using an enzyme-linked immunosorbent assay. The minimum detectable level was 5.0 pg/ml.

### Statistical analysis

Statistical significance was analyzed by using analysis of variance (ANOVA) followed by Bonferroni's t test post hoc to determine statistical differences. All values were expressed as means ± SD; p values < 0.05 were considered significant. The relationship between studied parameters was assayed by correlation. Pearson's linear correlation coefficient was used.

## Results

### Characteristics of patients and healthy volunteers are presented in table 1 (Table 1)

**Table 1:** Characteristics of study subjects and healthy volunteers.

Characteristics	Dimension	Healthy volunteers	Steroid naïve asthma	Stable ICS-treated asthma	Unstable ICS- treated asthma
Number of patients		12	14	16	16
Sex	F/M	6/6	8/6	9/7	10/6
Age	Years	25.40 ± 5.20	25.00 ± 6.30	39.50 ± 10.70	45.80 ± 7.30
Duration of symptoms	Years		2.71 ± 1.08^+Δ^	10.80 ± 6.20*^Δ^	17.06 ± 6.50*^+^
Baseline FEV_1_	% pred	102.50 ± 9.1^+Δ^	89.20 ± 12.00 ^Δ^	80.80 ± 7.10 ^Δ^	51.50 ± 11.70*^+^
FEV_1_/FVC ratio	%	86 ± 7	73 ± 8	64 ± 10	56 ± 12
Serum total IgE concentration	kU/L	61.08 ± 25.50*	248.4 ± 202.3	232.5 ± 79.0	318.0 ± 98.0
Blood eosinophil count	cells/mm^3^	56 ± 22*^+Δ^	212 ± 88	281 ± 73	302 ± 95
F_ENO_	ppB	15.80 ± 5.06*	75.21 ± 37.13^+^	39.40 ± 12.50*^Δ^	64.70 ± 25.04^+^
Eotaxin-1 (EBC)	pg/ml	6.24 ± 0.70*^+Δ^	9.70 ± 1.70^Δ^	10.45 ± 2.00^Δ^	17.97 ± 3.60*^+^
ECP (serum)	μg/l	3.87 ± 0.81*	13.21 ± 4.56	12.80 ± 3.50 ^Δ^	21.90 ± 8.40*^+^
ICS	μg/day			359 ± 128	1078 ± 269
Positive SPT					
mite/cat/moulds			14/2/3	14/4/4	15/4/6

Data are presented as medians (ranges)

FEV_1_- forced expiratory volume in one second

* Values significantly different from patients with steroid-naïve asthma, p < 0.05

^+ ^Values significantly different from patients with stable, ICS-treated asthma, p < 0.05

^Δ ^Values significantly different from patients with unstable, ICS-treated asthma, p < 0.05

ICS - inhaled corticosteroids (Fluticasone propionate equivalent)

SPT - skin prick tests (number of patients)

**Figure 1:**
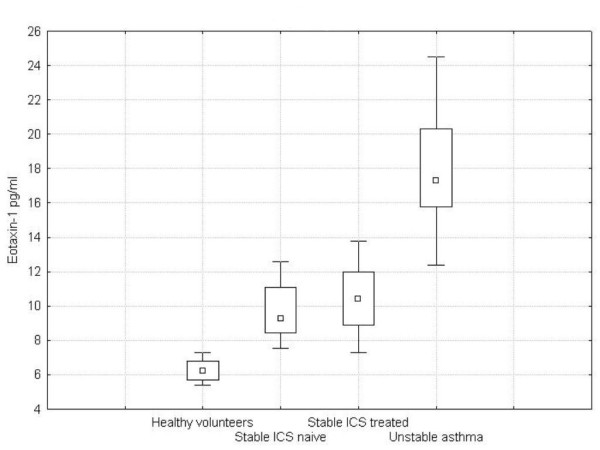
**Concentrations of eotaxin in EBC in studied groups of asthma patients and healthy volunteers**.

In the three groups of asthmatics, EBC concentrations of eotaxin-1 were significantly higher than those detected in healthy volunteers (steroid-naïve asthma: 9.70 pg/ml ± 1.70 [min. 7.56, max. 12.6], p = 0.002; ICS-treated stable asthma: 10.45 ± 2.00 [min. 7.3, max. 13.8], p < 0.001; unstable ICS-treated asthma: 17.97 ± 3.60 [min. 12.4, max. 24.5], p < 0.001; healthy volunteers: 6.24 ± 0.70 [min. 5.4, max. 7.3]) (Figure 1). Eotaxin-1 levels were elevated in patients with unstable ICS-treated asthma compared with ICS-treated stable asthma (p = 0.03) and steroid-naïve asthma patients (p = 0.009). We observed a tendency toward slightly lower eotaxin-1 concentrations in steroid-naïve asthma patients compared with the ICS-treated stable asthma group (p = 0.52).

**Figure 2:**
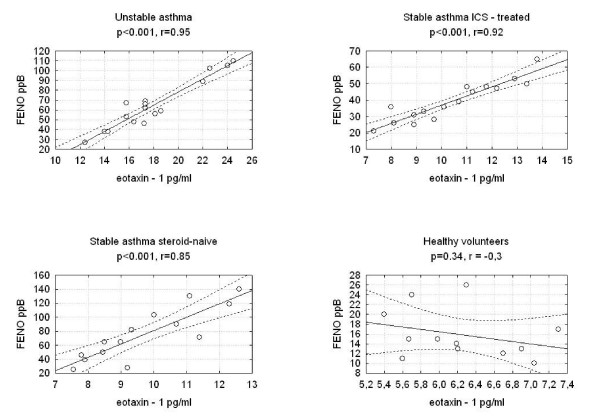
**Correlations between the eotaxin-1 levels and exhaled nitric oxide in studied groups of asthma patients and healthy volunteers**.

**Figure 3:**
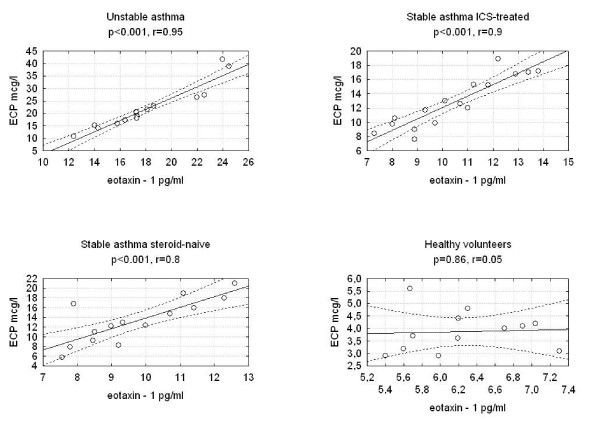
**Correlations between the eotaxin-1 levels and serum eosinophil cationic protein in studied groups of asthma patients and healthy volunteers**.

**Figure 4:**
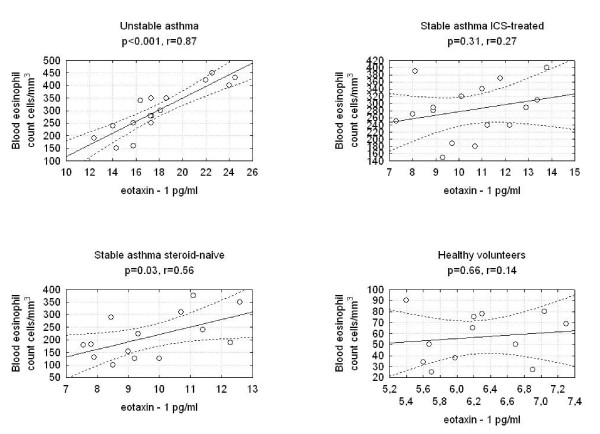
**Correlations between the eotaxin-1 levels and blood eosinophil count in studied groups of asthma patients and healthy volunteers**.

We found statistically significant correlations between the concentrations of eotaxin-1 in EBC and F_ENO _in the three studied groups of asthmatics. There were no correlations between eotaxin-1 in EBC and F_ENO _in the group of healthy volunteers (Figure 2). We discovered a significantly positive correlation between eotaxin-1 in EBC and serum ECP or blood eosinophil count in the groups of patients with unstable ICS-treated asthma and steroid-naïve asthma and between eotaxin-1 and serum ECP in the group of ICS-treated stable asthma (Figure 3, Figure 4). Statistically significant correlations between eotaxin-1 in EBC and other studied parameters were not observed in any studied groups of asthmatics or healthy volunteers (Table 2).

**Table 2:** Correlations between eotaxin concentrations in EBC and other studied parameters in the groups of asthma patients and healthy volunteers.

Studied groups	**F**_**ENO**_	Serum ECP	Blood eosinophil count	Serum total IgE	**Baseline FEV**_**1**_
Healthy volunteers	r = -0.30 p = 0.34	r = 0.05 p = 0.86	r = 0.14 p = 0.66	r = -0.34 p = 0.27	r = -0.52 p = 0.08

Steroid-naïve asthma	**r = 0.85** **p < 0.001**	**r = 0.80** **p < 0.001**	**r = 0.56** **p = 0.03**	r = -0.12 p = 0.66	r = -0.18 p = 0.53

Stable asthma ICS-treated	**r = 0.92** **p < 0.001**	**r = 0.90** **p < 0.001**	r = 0.27 p = 0.31	r = -0.17 p = 0.52	r = -0.31 p = 0.22

Unstable asthma ICS-treated	**r = 0.95** **p < 0.001**	**r = 0.95** **p < 0.001**	**r = 0.87** **p < 0.001**	r = -0.16 p = 0.54	r = -0.18 p = 0.48

**Figure 5:**
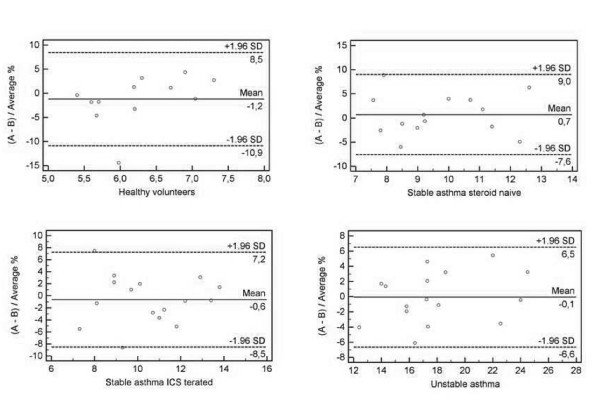
**Figure of differences in eotaxin-1 levels (measured in duplicates) against the mean, using Bland and Altman statistics in the studied groups of asthmatic patients and healthy volunteers**.

Differences in eotaxin-1 levels (measured in duplicates) against the mean, using Bland and Altman statistics in the studied groups of asthmatic patients and healthy volunteers are presented in figure 5.

## Discussion

Airway eosinophilia is recognized as a central event in the pathogenesis of asthma. The toxic components produced by eosinophils are thought to be important in inducing damage and dysfunction of bronchial mucosa [17]. Evidence suggests that recruitment of eosinophils into sites of inflammation is a multifractorial and multistep process, in which eosinophil-endothelial interactions through adhesion molecules, and local generation of chemotactic agents that direct cell migration into the inflamed airways, play an important role [18]. Therefore, adhesion molecules and chemokines are crucial mediators in selective eosinophil accumulation. In asthmatic patients, a relevant but variable correlation between blood eosinophilia and degree of asthma severity or bronchial hyperreactivity can be observed [19].

Eotaxin is the most specific and the strongest factor which can affect the function of eosinophil. The effect of eotaxin can be observed at each stage of the life cycle of eosinophil, and therefore plays a very important role in the development of allergic reaction. This chemokine is responsible for the release of progenitors of eosinophils from the bone marrow, and, together with IL-5, increases the count of mature forms in peripheral blood. The consequence of these processes is peripheral and tissue eosinophilia [20]. The count of eosinophils in an infiltrated organ is proportional to the concentrations of eotaxin in the site. Eotaxin is responsible for retardation of the apoptosis of eosinophils, and eotaxin interaction with a receptor leads to their activation and degranulation [2]. Eotaxin can also cause migration of mast cells [21] and basophils [22].

Those human cells which can produce eotaxin are the airway epithelium, endothelial cells, lymphocytes, macrophages, and eosinophils, as well as airway smooth muscle cells [2]. Eotaxin as a chemotactic factor for eosinophils plays an important role in the pathogenesis of asthma. It has been shown that eotaxin concentration in plasma correlates with the degree of bronchial hyperreactivity [23]. This concentration is also higher in asthmatics during exacerbation compared with patients with stable disease [24]. Higher levels of eotaxin in broncho-alveolar lavage (BAL), increased expression of mRNA for eotaxin, and an increase of eotaxin in bronchial epithelium [25] have been found in asthmatics compared with healthy volunteers.

In previous studies, the possibility of measuring of eotaxin levels in exhaled breath condensate was confirmed both in children [10] and in adults with asthma [9]. However, Leung et al did not detect any differences in eotaxin concentrations in EBC between groups of children with persistent asthma (on inhaled corticosteroids - ICS), intermittent asthma (without ICS) and healthy controls [10]. Ko et al demonstrated that eotaxin levels in EBC were higher in asthmatics than in controls, but the difference was no longer evident when, in analysis, steroid naïve mild asthma patients and healthy controls were taken into consideration [9]. It is worth noting that in any published study so far, correlations between eotaxin measurements in EBC and other parameters connected with airway inflammation - which seem to be necessary for recognizing the usefulness of this parameter in the assessment of inflammation - have not been uncovered.

The results of this study have confirmed the possibility of using eotaxin-1 measurements in EBC. It is worth noting that, in this study, increased eotaxin-1 levels in EBC in all groups of asthmatic patients with different degrees of disease severity compared with healthy volunteers were revealed for the first time. These differences could be the consequence of performing this study in highly selected groups and the authors have taken efforts to make this selection as defined as has been possible. By contrast, in the previouly cited studies, the differences between studied groups could have been too minor, and these studies were performed both on atopic and nonatopic patients and healthy volunteers.

This is the first report which demonstrates the differences in eotaxin-1 levels between patients with stable and unstable asthma and healthy volunteers. Our results are the first report in which it can be shown that concentrations of eotaxin-1 in EBC significantly correlate with exhaled nitric oxide levels - a more and more appreciable criterion for the evaluation of airway inflammation - in all studied groups of asthma patients [26-28]. Eotaxin-1 also correlates with other laboratory tests commonly associated with asthma, such as elevated levels of eosinophil cationic protein (all studied groups of asthma patients) and peripheral blood eosinophilia (unstable ICS-treated asthma and steroid-naïve asthma). Population studies indicate the presence of a connection between IgE concentrations and asthma or bronchial hyperreactivity. The results of our studies did not reveal any correlations between concentrations of eotaxin-1 in EBC and serum total IgE.

The results obtained here indicate the possibilities of wider use of eotaxin - 1 measurements in EBC in the assessment of airway inflammation. The correlations with other markers recognized in the evaluation of asthmatic inflammation suggest that, in this way, the possibilities of monitoring the course and treatment of asthma could be improved.

EBC examination, being simple and non-invasive, could be exploited to detect specific levels of biomarkers and monitor the severity of disease in response to appropriate prescribed therapy [15]. The analysis of EBC is still in the experimental phase. Many questions concerning the lack of standardization for both the collection and analysis of EBC, the repeatability of measurements, and the effect of many factors on concentrations of EBC markers, are still not answered. Reports from the EBC Task Force by the major American and European respiratory societies state that, although dilution may be a factor influencing EBC data, it does not appear to improve reproducibility. Because the marker used to correct the difference in the degree of dilution has not yet been established, in our study we have not taken attempts to assess the dilution of ALF in EBC. EBC volume does not depend on lung function parameters. There is no evidence to show that changes in airway caliber cause any difference in mediator release or dilution of EBC; however, this point is still under investigations. Cytokine concentrations in EBC are usually quantified by ELISA kits. Several different cytokines have been described to be present in EBC: IL 4, 6, 10, 1β, TNF-α [7,15]. However, the concentrations of several cytokines are around the lower limits of detection. EBC collection is a completely noninvasive way of sampling the respiratory tract with good reproducibility in EBC volume and mediator concentration for several markers: pH, H_2_0_2_, adenosine, 8-isoprostane [7,15].

Because of the difference in methodological procedures and the effect of many factors described previously, the results (different concentrations of eotaxin-1) of our study may not be directly comparable with the results from other research groups. We suggest that in the assessment of measurements of concentrations of immunological markers, including chemokines, in EBC, the control group and analysis of observed changes between the studied groups, as well as changes in the studied parameters in time, should be taken into account.

In contrast to our previous studies, in which we suggested the beneficial effect of inhaled corticosteroids treatment on downregulation of RANTES in the airways (using EBC) [29], analysis of the results of this study does not indicate the similar effect of ICS-treatment on eotaxin-1 levels in EBC. Similar observations were published by Ko et al, which revealed that subjects on high-dose ICS had similar eotaxin levels in EBC when compared with patients on low-to-moderate doses of ICS [9]. Feltis et al did not reveal the effects of three-month treatment with ICS on eotaxin levels in BAL [4]. Similarly, Tateno et al noted that the plasma eotaxin level was not altered by inhaled or oral corticosteroid treatment [30]. However, *in vitro *studies have demonstrated that dexamethasone inhibition of cytokine-induced eotaxin mRNA augmentation is associated with diminished eotaxin secretion in cell cultures [31]. Further studies are needed for a better understanding of the effect of ICS on eotaxin in the asthmatic patients.

There are some limitations of the study. One of them is small number of patients in the studied groups. The number of patients in particular groups was based on our experiences from previous studies, in which in similar numbers of patients the possibility of obtaining statistically significant differences in studied parameters in EBC, as well as in peripheral blood, was revealed. Because of the small sizes of our studied groups, an analysis of the minimal number of the sample using statistical tests was not performed. The next limitation of our study worth noting is the difference in age between the studied groups of asthmatics and the healthy volunteers. These differences between studied groups are a consequence of the natural history of asthma, diagnosis at a young age, and the subsequent, sometimes severe, course of the disease. Previous studies published by Targowski et al have shown that age and sex significantly influence the serum eotaxin levels in healthy people and patients with rhinoconjunctivitis [32]. However, in the authors' opinion, the differences in age observed in this study between unstable ICS-treated asthma and stable ICS-treated asthma, as well as between steroid-naïve asthma and healthy volunteers, are small and irrelevant. Moreover, observed differences in eotaxin -1 levels could be rather the consequence of intensification of the inflammatory process, not of differences in age, and correlate with other markers of airway inflammation.

In conclusion, we have shown that eotaxin-1 levels in exhaled breath condensate are higher in asthmatic patients with different degrees of asthma severity when compared with controls. In patients with unstable asthma, these values are significantly higher compared with subjects with stable disease and correlate with other inflammatory parameters such as exhaled nitric oxide or serum ECP. Measurements of eotaxin-1 in EBC of asthma patients may provide another useful diagnostic tool for detecting and monitoring airway inflammation. However, taking the previously described methodological limitations of our study into account, future studies are needed for better assessment of the clinical significance and the possibility of the practical usefulness of eotaxin-1 measurements in EBC.

## Competing interests

The authors declare that they have no competing interests in the publication of the manuscript. This work was supported by research grant No 3-06513P from the Medical University of Bialystok, Poland.

## Authors' contributions

ZZ conceived the trial, participated in its design, study procedures, interpretation of results, performed the statistical analysis and helped to draft the manuscript. MMT-L participated in the study procedures and helped to draft the manuscript. RS participated in the study procedures, laboratory tests and helped to draft the manuscript. EZ helped to draft the manuscript. AB-L participated in study design, interpretation of results and helped to draft the manuscript. All of the authors read and approved the final manuscript.
